# Performance enhancement of wood composites using cellulose-reinforced cornstarch–tannin adhesives derived from electrical-assisted extraction

**DOI:** 10.1039/d5ra10055k

**Published:** 2026-02-12

**Authors:** Yassine El Khayat Driaa, Hafida Maarir, Nabil Grimi, Nadia Boussetta, Amine Moubarik

**Affiliations:** a Integrated Transformation and Renewable Matter TIMR (UTC/ESCOM), University of Technology of Compiegne – Alliance Sorbonne University, Centre of Research of Royallieu Rue du docteur Schweitzer, CS 60319 60203 Compiegne France yassine.el-khayat-driaa@utc.fr; b Chemical Processes and Applied Materials Team, Polydisciplinary Faculty, Sultan Moulay Slimane University SBP: 592 Beni Mellal Morocco a.moubarik@usms.ma

## Abstract

This study investigates the impact of electrical-assisted extraction techniques on cellulose derived from almond shells and its performance in formaldehyde-free cornstarch–mimosa tannin (CM) adhesives. Cellulose was extracted using three methods: conventional alkali treatment (AT), alkali treatment assisted by pulsed electric fields (PEF), and alkali treatment assisted by high-voltage electrical discharges (HVED). Comprehensive analyses, including FTIR, XRD, TGA/DTG, DSC, and SEM, were conducted to evaluate the chemical structure, crystallinity, thermal stability, and morphology of the extracted celluloses. The results revealed that while cellulose yield varied slightly among treatments, electrical-assisted extraction significantly enhanced delignification and fibrillation without altering the cellulose I crystalline structure. Both PEF- and HVED-treated celluloses exhibited improved crystallinity (≈59%) and thermal stability (*T*_onset_ ≈ 303 °C), indicating superior structural integrity. Incorporation of these celluloses into CM adhesives increased viscosity, solid content, and shear strength, with optimal performance at 6 wt% cellulose loading. Particleboards bonded with HAC-CM adhesives showed the highest mechanical properties (IB = 0.79 MPa, MOR = 32.27 MPa, MOE = 3125 MPa), exceeding EN 312 (P4) standard requirements. Water absorption and thickness swelling were markedly reduced, confirming enhanced moisture resistance. Overall, HVED-assisted extraction produced cellulose with superior reinforcing capability, demonstrating a sustainable and high-performance pathway for developing formaldehyde-free wood adhesives from agricultural residues.

## Abstract 2

High-voltage electrical extraction transforms almond shell waste into reinforcing cellulose for starch–tannin adhesives. The resulting formaldehyde-free particleboards exhibit enhanced mechanical properties, meeting EN 312 standards.

## Graphical abstract

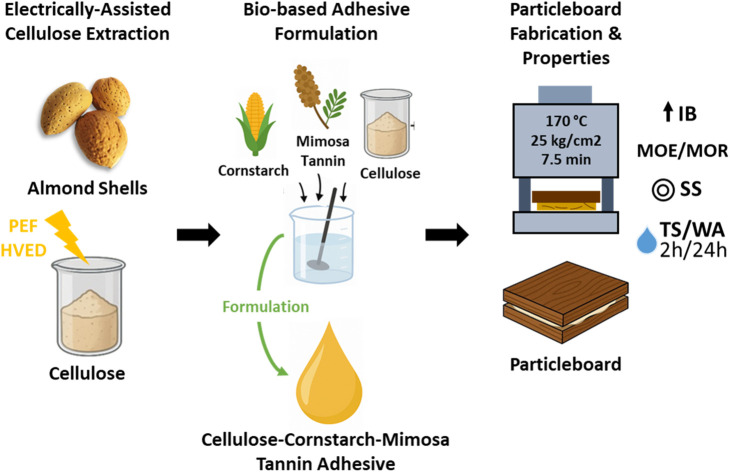

## 1 Introduction

Formaldehyde-based adhesives have long dominated the wood panel industry due to their superior bonding performance and cost-effectiveness. However, growing awareness of their adverse health and environmental effects, particularly the emission of formaldehyde, a known human carcinogen, has led to increasing regulatory scrutiny and consumer concern.^1,2^ In response, considerable research efforts have shifted toward the development of formaldehyde-free, bio-based adhesives utilizing renewable natural polymers such as cornstarch, tannins, lignin, and cellulose.^3–6^ Despite the promise of bio-based adhesives, a significant technical bottleneck remains: their inherent sensitivity to moisture. Traditional carbohydrate-based adhesives, such as pure starch, rely heavily on a network of hydrogen bonds.^7^ These bonds are easily disrupted upon water adsorption, leading to high thickness swelling and a rapid loss of interfacial bond strength. Current formaldehyde-free alternatives often fail to provide the necessary hydrophobic barrier or the cross-linking density required to match the durability of synthetic resins. Addressing this limitation requires a strategy that not only reinforces the matrix but also preserves the chemical reactivity of the bonding agents during the extraction process.^1,4^ Recent studies have also demonstrated the effectiveness of tannin-based adhesive systems relying on physical crosslinking and hydrogen-bonding interactions for wood bonding. For example, Chen *et al.* reported a physically crosslinked tannic acid–based adhesive exhibiting strong adhesion to wood and good biocompatibility, highlighting the key role of polyphenol-mediated intermolecular interactions.^8^ These findings are consistent with the interaction mechanism and performance trends observed in the present starch–tannin–cellulose adhesive system.

Cellulose, the most abundant biopolymer on Earth, is increasingly recognized for its valuable role in bio-adhesive formulations due to its high mechanical strength, thermal stability, and biodegradability.^9,10^ Its crystalline structure contributes to adhesive durability and heat resistance, while its renewable nature supports environmental goals by reducing reliance on synthetic, formaldehyde-based adhesives.^11,12^ When combined with other natural polymers such as cornstarch and mimosa tannins, cellulose reinforces adhesive networks through hydrogen bonding and phenolic interactions, improving both bonding strength and water resistance.^13^ However, the efficiency of cellulose as a reinforcing agent depends heavily on its extraction process and structural preservation. Challenges remain in improving water resistance and simplifying processing steps, highlighting the need for further innovation in cellulose extraction and bio-adhesive formulation.^10,12^

Almond shells, a lignocellulosic by-product of the almond industry, represent a valuable and underutilized biomass resource for cellulose extraction due to their high fiber content and widespread availability.^14^ Conventional extraction approaches, such as alkali treatment, typically rely on harsh chemical agents, elevated temperatures, and prolonged reaction times, which not only pose environmental concerns but also risk degrading cellulose structure and lowering extraction yields.^15,16^ These methods are often inefficient, requiring multiple processing steps and generating significant chemical waste. In contrast, electrical-assisted extraction techniques, particularly PEF and HVED, have emerged as promising green alternatives. PEF operates by applying short, high-voltage pulses to induce electroporation and enhance cell membrane permeability, thereby facilitating the release of intracellular materials with reduced chemical input and energy consumption.^17^ HVED, a non-thermal method, uses plasma generation and shockwaves to disrupt biomass structure more intensively, improving cellulose liberation while preserving its functional integrity.^17^ The adoption of these technologies aligns with the principles of sustainable processing and offers solutions for cellulose extraction from agricultural residues.^16^ This study addresses the limitations of traditional methods by employing EAE to isolate alkali cellulose, hypothesizing that EAE-induced electroporation facilitates a gentler, more efficient removal of non-cellulosic components. By preserving the structural integrity and increasing the surface hydroxyl accessibility of the fibers, this specific modification is expected to enhance the ‘bridging effect’ and mechanical interlocking within the cornstarch–tannin matrix. Drawing on recent advancements in electro-technologies for biomass valorization,^18–20^ this work highlights how preserving fiber quality through EAE creates a superior reinforcing ‘skeleton,’ ultimately overcoming the traditional trade-off between bio-based sustainability and industrial bond strength.

This study aims to evaluate and compare the effects of different cellulose extraction methods, alkali treatment, PEF combined with alkali treatment, and HVED combined with alkali treatment, on the structural and functional properties of cellulose derived from almond shells. The extracted cellulose samples were thoroughly characterized using FTIR, XRD, TGA/DTG, DSC, and SEM to assess changes in chemical structure, crystallinity, thermal behavior, and morphology. The study further investigates the influence of extraction technique on the performance of formaldehyde-free bio-adhesives formulated with cellulose, cornstarch, and mimosa tannins. Adhesive performance was evaluated through key mechanical and physical tests, including dry internal bond (IB), modulus of elasticity (MOE) and rupture (MOR), shear strength (SS), thickness swelling, and water absorption. By correlating the extraction method with both cellulose quality and adhesive performance, this work seeks to identify high-efficiency pathways for producing sustainable, high-performance wood adhesives from agricultural waste.

## 2 Materials and methods

### 2.1 Materials and chemicals

The almond shells (AS) utilized in this study were sourced from local cooperatives in Zagora, located in the Drâa-Tafilalet region of southern Morocco. These agricultural by-products underwent rigorous preparation, including thorough triple washing, sun-drying, crushing, and careful storage before use. All chemicals employed in this research were procured from Merck (Sigma–Aldrich, France).

### 2.2 Raw material composition

The moisture content was determined using the Infrared Moisture Analyzer MA160 from Sartorius, Germany. Ash content was determined by incinerating 2 g of ground almond shells in a Thermo Fisher Scientific Thermolyne FB1315M muffle furnace at 550 °C for 6 hours. The residue was weighed after cooling, and ash content was calculated as the percentage of inorganic material relative to the initial sample weight. Extractives and Klason lignin contents were quantified following to the Laboratory Analytical Procedure (LAP) established by the National Renewable Energy Laboratory (NREL).^21^ For the extraction process, approximately 3–4 g of dry powder were placed in a cellulose cartridge positioned above a 500 mL flask containing 300 mL of dichloromethane. Extraction was carried out over 8 hours. The solvent was then removed using a rotary evaporator at 40 °C to recover the extractives. The flask with the extractive and the treated powder was subsequently dried in an oven at 105 °C for 24 hours and weighed after cooling. The treated powder was hydrolyzed with 72% sulfuric acid for 1 hour, followed by dilution with water to achieve a 4 wt% sulfuric acid concentration. The diluted sample was autoclaved and then filtered. The dried residue was weighed to determine the Klason lignin content. All analyses were performed in triplicate, and standard deviations were calculated to ensure accuracy and reproducibility.

### 2.3 Cellulose extraction

#### 2.3.1 Alkali extraction

**Fig. 1:**
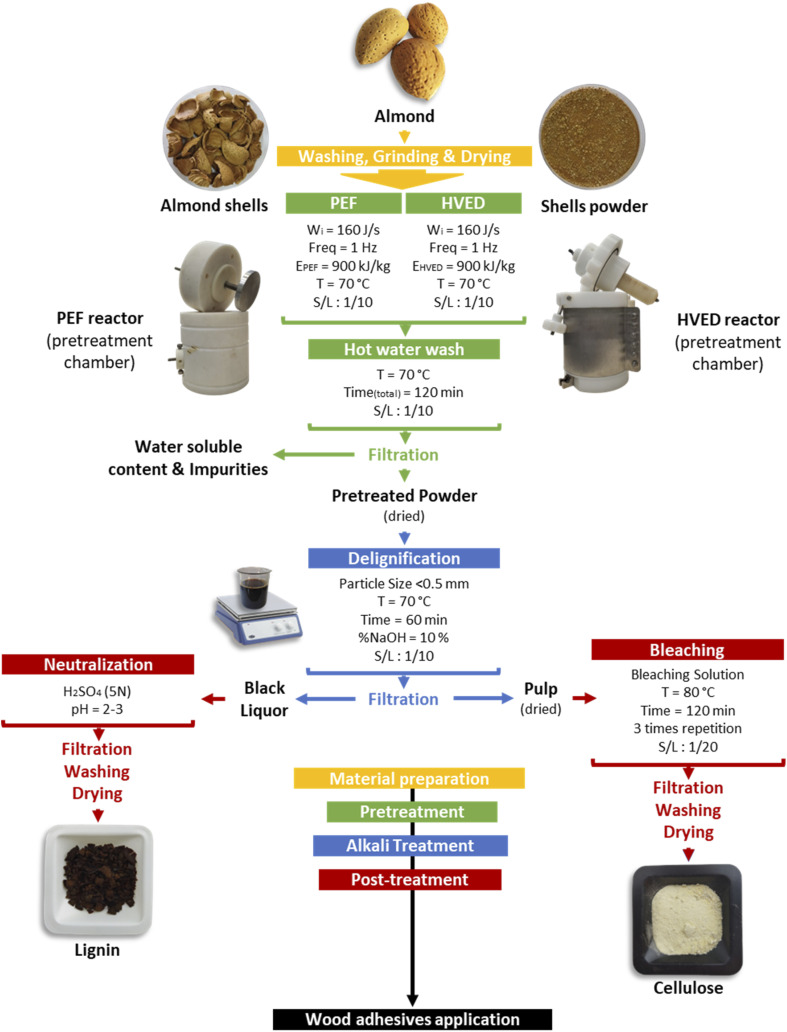
Schematic overview of cellulose extraction from almond shells using PEF and HVED-assisted alkaline treatment.

Control conditions for almond shells were established through alkaline treatment, following the methods (Fig. 1) described previously.^4,9,22^ Initially, 5 g of raw material were ground and sieved to obtain three distinct particle size fractions (1–0.75 mm, 0.75–0.5 mm, and <0.5 mm). These samples were then separately washed with hot water at 70 °C for 2 hours to eliminate partially water-soluble materials and impurities. Subsequently, the washed samples underwent alkaline treatment at temperatures of 50, 70, 90, and 98 °C for 30, 60, 90, and 120 minutes, respectively. Aqueous sodium hydroxide (NaOH) solutions at different concentrations (5%, 10%, 15%, and 20% w/v) were employed to achieve optimal extraction conditions. The solid to liquid ratio was maintained at 1 : 10 (w : v). After alkali extraction, the suspension was filtered to separate the solid (pulp) from the liquid (black liquor) parts. The resulting pulp underwent bleaching at 80 °C for 2 hours using a bleaching solution containing acetate buffer (27 g NaOH and 75 mL glacial acetic acid, diluted with 1 L of distilled water) and 1.7 wt% NaClO2 in water. The solid-to-liquid ratio for bleaching was 1 : 20 (w : v). To ensure further removal of amorphous components, the bleaching process was repeated twice. The cellulose obtained post-bleaching was washed with distilled water and oven-dried, resulting in alkali-treated cellulose (AC). Sulfuric acid was introduced into the obtained black liquor until reaching a pH of 2–3 to precipitate acidified lignin. Subsequently, the separated lignin underwent thorough washing until a neutral pH was reached. Finally, the obtained wet lignin was oven-dried, resulting in alkali-treated lignin. Three replicates were conducted for each material and concentration during the treatment, and standard deviations were calculated accordingly.

The yields of cellulose and lignin were determined in accordance with eqn (1):1
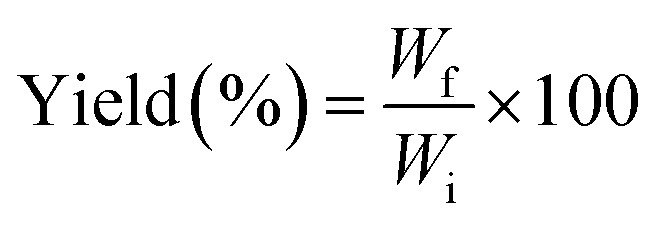
where *W*_i_ is the initial total dry weight of the raw materials (g) and *W*_f_ is the final dry weight of the isolated cellulose or lignin (g).

#### 2.3.2 Electrical pretreatment

The electrical pretreatment conditions were established based on methods previously described in the literature^9,23^ (Fig. 1). In this study, a specific energy input of 900 kJ kg^−1^ was selected, guided by preliminary experimental results.^9^ For each treatment, 25 g of raw material was introduced into the treatment chamber. Preheated water at 70 °C was then added, maintaining a solid-to-liquid ratio of 1 : 10 (w/v).

The equipment employed for PEF treatment consisted of a pulsed high-voltage power supply (Basis, Saint-Quentin) and a 1 L processing chamber. The chamber was equipped with two parallel disk electrodes, each with a diameter of 11 cm. The generated pulses had a duration of 10 µs and a frequency of 1 Hz. The generator could produce exponential decay pulses, with a maximum voltage output of 40 kV and a peak current of 10 kA.

The specific energy input (E, kJ kg^−1^) for PEF treatment was determined according to eqn (2):2
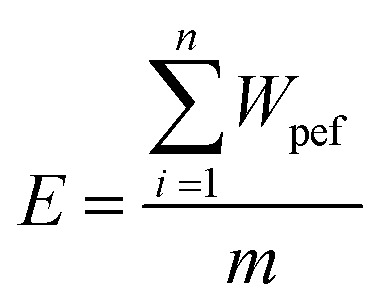
In this context, WPEF represents the energy per pulse (kJ per pulse), *n* is the pulse number, and *m* denotes the suspension mass (kg).

The determination of *W*_PEF_ is based on eqn (3):3
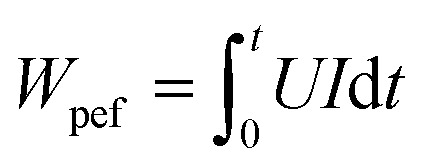
where *U* represents the voltage (V) and *I* represents the current (*A*).

The processing energy applied was 900 kJ kg^−1^. The total PEF application time, *t*_pef_, (s) was calculated as the product of the mean pulse width, ti, (s) and the number of pulses *n*_pef_ as follows eqn (4):

4 *t*_pef_ = *n*_pef_ × *t*_i_

The overall duration of solid–liquid interaction throughout the PEF treatment is given by eqn (5):5
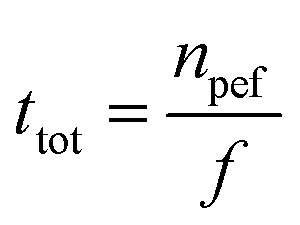
where *f* represents the pulse frequency (s^−1^).

HVED experiments utilized the same generator as the PEF setup, with the primary difference being electrode geometry. The chamber was equipped with rod-plate geometry electrodes. The stainless-steel rod electrode had a diameter of 10 mm, while the grounded disk electrode featured a diameter of 35 mm. The inter-electrode distance was fixed at 0.5 cm. A positive discharge voltage of 40 kV was applied to the rod electrode as the peak discharge voltage (U).

The specific energy input (E, kJ kg^−1^) for the HVED treatment was determined using the following eqn (6):6
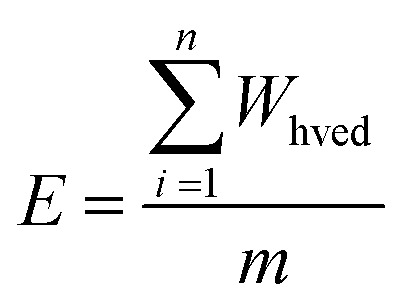
In this context, *W*_HVED_ represents the pulse energy per discharge (kJ per discharge), and *m* denotes the suspension mass (kg).

The determination of *W*_HVED_ is based on the eqn (7):7
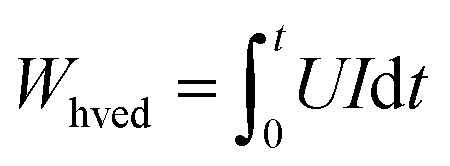
where *U* represents the voltage (V) and *I* represents the current (*A*).

The processing energy applied was 900 kJ kg^−1^. The HVED treatment time, *t*_hved_, (s) was calculated as the product of the mean discharge width, *t*_i_, (s) and the number of pulses *n*_hved_ as follows eqn (8):

8 *t*_hved_ =*n*_hved_ ×*t*_i_

The overall duration of solid–liquid interaction throughout the HVED treatment is given by eqn (9):9
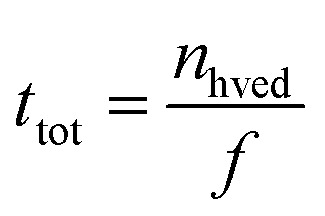
where *f* represents the pulse frequency (s^−1^).

After each physical pretreatment, the alkali treatment was performed.

### 2.4 Cellulose characterizations

#### 2.4.1 Structure and crystallinity analysis

In this study, the Nicolet Summit LITE FTIR Spectrometer was used to analyze and identify the characteristic functional groups of each sample. The samples were finely ground and mixed with dried potassium bromide (KBr) at a 1 : 100 sample-to-KBr ratio to prepare pellets. The analysis was performed over a frequency range of 500–4000 cm^−1^, with a resolution of 0.6 cm^−1^, and 16 scans were averaged for each measurement.

The crystalline structure of all cellulosic samples was analyzed using an X-ray diffractometer (BRUKER Diffractometer, D8 ADVANCE). Each material was ground into powder and uniformly placed on the sample holder to ensure consistent X-ray exposure. The samples were scanned using Cu Kα radiation (*λ* = 1.5406 Å) over a 2*θ* range of 5° to 40°, with the instrument operated at a voltage of 40 kV and a current of 40 mA. To evaluate the crystallinity of the cellulosic materials, the crystallinity index (CrI) was calculated using Segal's eqn (10):^24^10
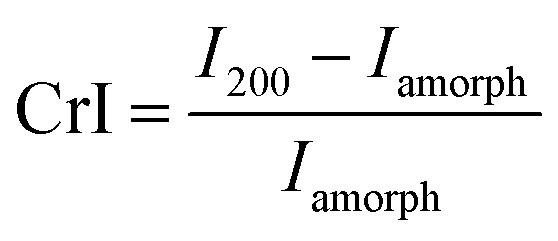
where, *I*_200_ represents the maximum intensity of the crystalline peak at around 2*θ* = 22.8°, and *I*_amorph_ is the intensity from the amorphous phase at approximately 2*θ* = 18.6°.

#### 2.4.2 Thermal stability analysis

Thermogravimetric analysis (TGA/DTG) and differential scanning calorimetry (DSC) were carried out using the Setaram 131 EVO instrument. For each analysis, 25 mg of sample was subjected to a controlled heating process from 30 °C to 600 °C at a heating rate of 5 °C min^−1^ under a nitrogen atmosphere with a flow rate of 20 mL min^−1^. The TGA/DTG analysis provided insights into the weight loss profile of the sample as a function of temperature, while the DSC analysis measured heat flow, capturing phase transitions and chemical reactions occurring within the sample.

#### 2.4.3 Morphology analysis

The impact of electrical pretreatment and chemical treatment on the morphology of cellulose was investigated using a scanning electron microscope (SEM, FEI, QUANTA FEG 250) operating at an acceleration voltage of 20 kV. Specimen preparation involved coating the samples with a thin layer of conductive carbon using an ion-sputtering device to ensure proper electron conductivity during imaging.

### 2.5 Cellulose-based wood adhesive formulation

**Fig. 2:**
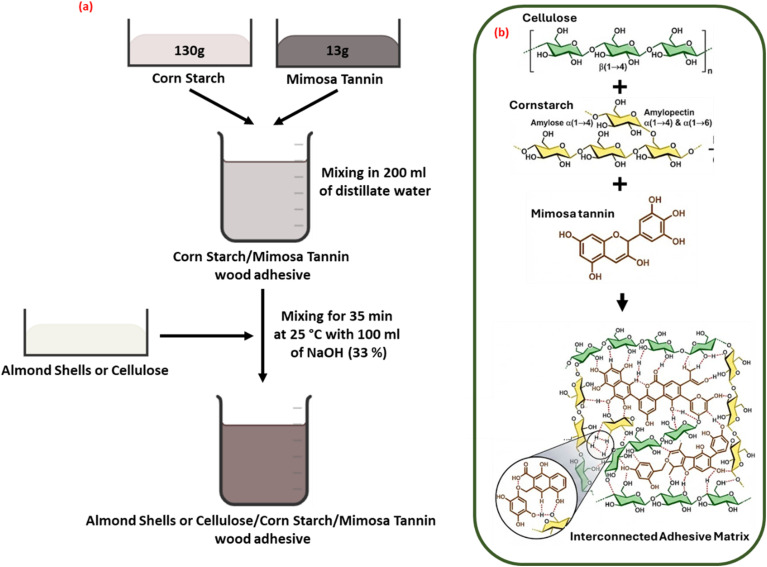
(a) Formulation process and (b) illustration of the plausible adhesion mechanism between cellulose-corn starch-mimosa tannin adhesives.

The adhesive formulations were prepared at room temperature by incorporating varying proportions (0, 2, 4, 6, and 8 w/w) of almond shell powder or extracted cellulose into a previously developed cornstarch–mimosa tannin (CM) bio-adhesive formulation, following methods described in earlier studies^3,25,26^ (Fig. 2). Initially, 130 g of corn starch and 13 g of mimosa tannin were dissolved in 200 mL of distilled water under continuous mechanical stirring at room temperature. A separate 30% hexamine solution was prepared and subsequently added to the CM mixture. The different formulations were obtained by adjusting the almond shell powder or extracted cellulose content according to the target proportions. Sodium hydroxide solution (33%) and the hexamine solution were then added to each formulation, and the mixtures were stirred for 35 minutes to ensure complete homogenization. The resulting adhesives were immediately used to fabricate particleboard panels.

### 2.6 Particleboard preparation

Particleboards were fabricated following the procedure described in previous studies.^3,25,26^ Particleboards with dimensions of 350 mm × 350 mm × 14 mm were manufactured using a hot-pressing method at 170 °C under a pressure of 25 kg cm^−2^ for 7.5 minutes. The adhesive system consisted of a cornstarch–mimosa tannin (CM) formulation modified by incorporating different proportions (0, 2, 4, 6, and 8 w/w) of almond shell powder or extracted cellulose. A total adhesive solids content of 9% by weight was maintained for all formulations. After hot pressing, the particleboards were initially conditioned at 25 °C and 65% relative humidity for 24 hours in a Vötsch climate chamber, followed by further conditioning at approximately 28 °C and 65% relative humidity for five days to stabilize internal stresses and achieve a final moisture content close to 10%. The target density for the particleboards was set between 650 and 750 kg m^−3^. Five particleboards were produced for each adhesive formulation, including the control without almond shell powder or cellulose.

### 2.7 Particleboard characterization

Particleboard specimens were rigorously tested in accordance with established European Standards to comprehensively evaluate their mechanical and physical performance. Mechanical properties assessed included dry internal bond strength (IB) following EN 319 (1993), modulus of rupture (MOR) and modulus of elasticity (MOE) as per EN 310 (1993), and surface soundness (SS) according to EN 311 (1993). To assess durability and dimensional stability, water absorption (WA) and thickness swelling (TS) were measured after 2 and 24 hours of immersion at room temperature, capturing both immediate and prolonged effects in accordance with EN 317 (1993). Measurements of weight gain and thickness were recorded immediately post-immersion. Each test was performed on triplicate particleboard samples bonded with CM adhesives at varying weight ratios, enabling evaluation of adhesive formulation impact on board performance.

### 2.8 Statistical analyses

Statistical analysis was performed using Two-way analysis of variance (ANOVA) in OriginLab software (version 2019b 9.65, Massachusetts, USA) with a significance threshold of 5%. For each analysis, a Tukey test was employed as a comparative method to evaluate the significance of the observed differences in the results.

## 3 Results and discussions

### 3.1 Chemical composition of raw materials

The chemical composition of almond shells (Table 1) varies significantly by geographic origin, reflecting differences in environmental conditions, cultivar types, and post-harvest processing. In this study, Moroccan almond shells were found to have a relatively high lignin content (34.9 ± 0.7 wt%), comparable to or exceeding values reported for other regions such as Tunisia (30.1 ± 0.5 wt%) and India (31.7 wt%), and notably higher than those from Spain (21.2 ± 2.0 wt%). The extractive content in Moroccan almond shells (11.3 ± 0.2 wt%) was also similar to Tunisian samples (11.8 ± 0.2 wt%) but lower than that observed in Spanish shells (16.3 ± 1.5 wt%), indicating a moderate presence of non-structural components. Although cellulose and hemicellulose values were not determined in the Moroccan sample, literature values from other regions ranged widely, with the highest cellulose content reported for Turkish shells (50.7 wt%) and the highest hemicellulose content in Iranian shells (35.3 wt%). The ash content of Moroccan shells (1.3 ± 1.0 wt%) was among the lowest, suggesting a lower mineral load compared to most other origins, such as Tunisia (3.4 ± 0.1 wt%) and Turkey (3.3 wt%). Overall, the Moroccan almond shells analyzed in this work present a lignin-rich and low-ash biomass profile, making them a promising raw material for lignocellulosic biomass valorization.

**Table 1:** Comparative chemical composition of almond shells from different geographic origins

Almond shells origins	Moisture	Cellulose	Hemicellulose	Lignin	Extractives	Ash	References
	wt%						
Morocco	9.2 ± 0.1	—	—	34.9 ± 0.7	11.3 ± 0.2	1.3 ± 1.0	This work
Tunisia	—	29.9 ± 0.7	25.1 ± 0.7	30.1 ± 0.5	11.8 ± 0.2	3.4 ± 0.1	27
Spain	—	26.8 ± 1.3	23.6 ± 0.2	21.2 ± 2.0	16.3 ± 1.5	2.0 ± 0.2	28
Turkey	—	50.7	28.7	20.4	—	3.3	29
Iran	3.0	29.1	35.3	32.7	—	3.4	30
India	5.0	32.5	29.5	31.7	—	3.1	14
China	—	38.5	28.8	29.5	8.0	—	31

### 3.2 Cellulose extraction

#### 3.2.1 Effects of alkali treatment parameters

##### 3.2.1.1 Effect of sample particle size

**Fig. 3:**
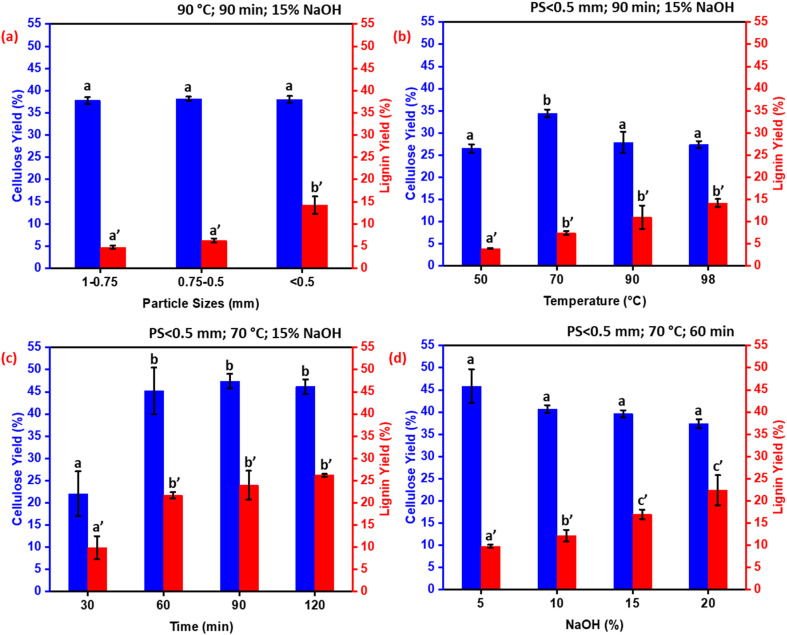
Effect of particle size (a), extraction temperature (b), extraction time (c), and NaOH concentration (d) on the yields of cellulose and lignin extracted from almond shells.

Fig. 3a depicts the effect of particle size on the extraction yields of cellulose and lignin. Almond shells were subjected to alkali treatment following conditions: 90 °C, 90 min, using 15% of NaOH. The results showed that particle size has no significant effect on cellulose yield. For lignin, the yield increases when the particle size is reduced from 0.75–0.5 mm to <0.5 mm. This can be attributed to the increased specific surface area of smaller particles, which enhances solvent and reagent penetration, thereby improving lignin extraction efficiency.^32^ The hard and dense structure of almond shells, predominantly composed of lignin, provides substantial mechanical strength, making it difficult to access internal compounds during extraction. Thus, optimizing particle size is crucial for enhancing extraction efficiency. Reducing particle size increases the surface area available for chemical reactions and solvent interaction, facilitating the breakdown of cell wall components.^33^ For cellulose, bleaching was insufficient for particles sized between 1 mm and 0.75 mm, likely due to limited reagent penetration and surface accessibility. Therefore, an optimal particle size below 0.5 mm was selected to ensure effective extraction and bleaching efficiency.

##### 3.2.1.2 Effect of extraction temperature

Fig. 3b shows the effect of extraction temperature on the extraction yields of cellulose and lignin. Almond shells were subjected to an alkali treatment with the following conditions: particle size <0.5 mm, 90 min, using 15% of NaOH. The cellulose yield increases when the extraction temperature rises from 50 °C to 70 °C, but decreases when the temperature goes from 70 °C to 98 °C. The highest yield was obtained at 70 °C. For lignin, the yield increases slightly from 50 °C to 70 °C, with no significant change between 70 °C and 98 °C. Therefore, 70 °C was considered as the optimal extraction temperature.

##### 3.2.1.3 Effect of extraction duration

Fig. 3c presents the effect of extraction duration on cellulose and lignin yield. Almond shells were subjected to alkali treatment with the following conditions: particle size of <0.5 mm, 70 °C, and using 15% of NaOH. The results showed that the cellulose yield increased with extraction time, following an exponential trend. A saturation plateau was observed after 60 minutes. For lignin, the results showed that the lignin yield increases following an exponential trend. However, beyond 60 minutes, the lignin yield reaches a plateau, suggesting that further extending the extraction time does not significantly enhance yield. This plateau indicates that an extraction time of around 60 minutes is optimal for lignin extraction, balancing efficiency and practicality without unnecessarily prolonging the process. Furthermore, lignin's structure and solubility can be altered over extended extraction periods, influencing the overall extraction process.^34^

##### 3.2.1.4 Effect of sodium hydroxide concentration

Fig. 3d illustrates the effect of NaOH concentration on the extraction yields of cellulose and lignin. Almond shells were subjected to alkali treatment with the following conditions: particle size <0.5 mm, 70 °C for 60 minutes. For lignin, a significant increase in extraction yield is observed when the NaOH concentration increased from 5% to 10%. Beyond this point, higher alkali concentrations did not produce a notable improvement in yield. The results suggested that a 10% NaOH concentration was optimal for extracting cellulose and lignin, balancing efficiency and reagent consumption. These findings highlight the importance of optimizing NaOH concentration to maximize lignin extraction while minimizing the detrimental effects on cellulose yield. During the extraction process, NaOH dissolves lignin and hemicellulose, forming soluble complexes,^34^ while the relatively lower reactivity of cellulose toward NaOH allows it to remain structurally intact and preserved.

The previous analysis of the alkali treatment parameters (particle size, temperature, duration, and NaOH concentration) established the optimal conditions for maximizing cellulose and lignin extraction from almond shells: PS < 0.5 mm, 70 °C, 60 min, and 10% of NaOH. However, these optimal conditions were fixed for the subsequent material treatment section, Section 2.2.2 (electrical pretreatment). This fixed set of robust alkali conditions ensures that the structural integrity of the almond shell is sufficiently compromised, providing a standardized and activated material for a focused investigation into the unique effects of the electrical pretreatment.

#### 3.2.2 Effect of electrical pretreatment on cellulose extraction

##### 3.2.2.1 Effects on cellulose yield

**Fig. 4:**
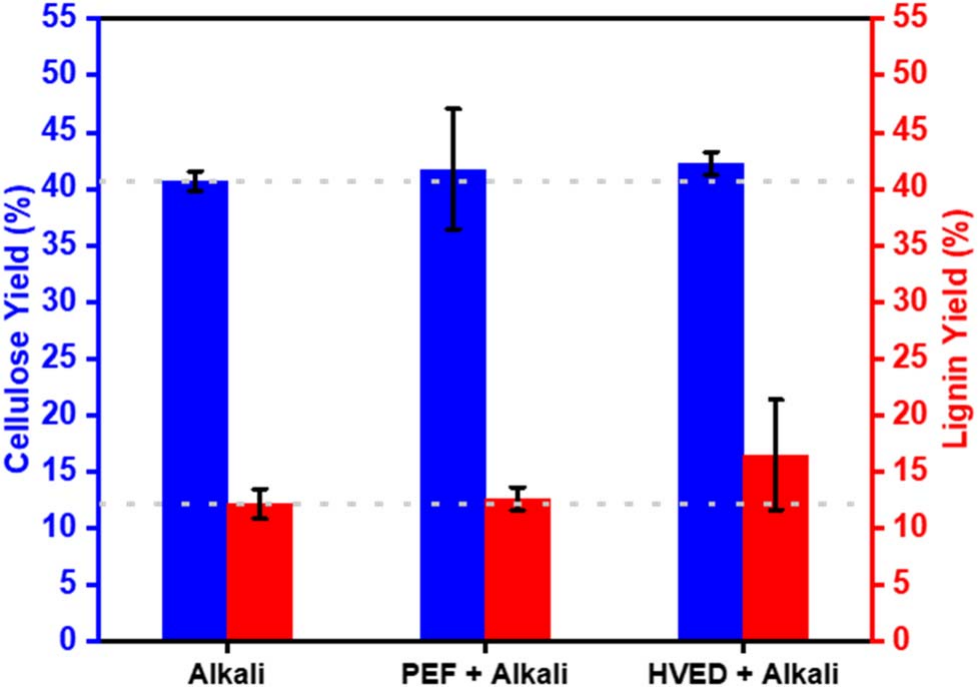
Influence of PEF and HVED on the yields of cellulose and lignin extracted from almond shells.

The cellulose yields recovered from almond shells (Fig. 4) *via* conventional alkali treatment (AT: 40.68 ± 0.87 wt%) were found to be comparable to those obtained using PEF-assisted (PAC: 41.72 ± 5.32 wt%) and HVED-assisted extraction (HAC: 42.24 ± 2.95 wt%). Statistical analysis confirmed that these 1–2% variations are not significant (*p* > 0.05), suggesting that under the specific conditions applied (900 kJ kg^−1^, 1 Hz, 70 °C), electrical pretreatments do not substantially increase the total mass recovery of cellulose. This outcome is likely due to the highly lignified and dense structural matrix of almond shells, which limits the incremental yield potential of additive physical treatments.^31,35^ However, the primary value of Electrical-assisted extraction in this context is not the enhancement of yield quantity, but rather the potential for structural modification. While mass recovery remains stable across all methods, the electrical discharge may promote surface fibrillation and hydroxyl accessibility—factors that are more critical than yield for the reinforcing performance of the cellulose in bio-based adhesive applications.^9,36^

##### 3.2.2.2 Structure and crystallinity analysis

**Fig. 5:**
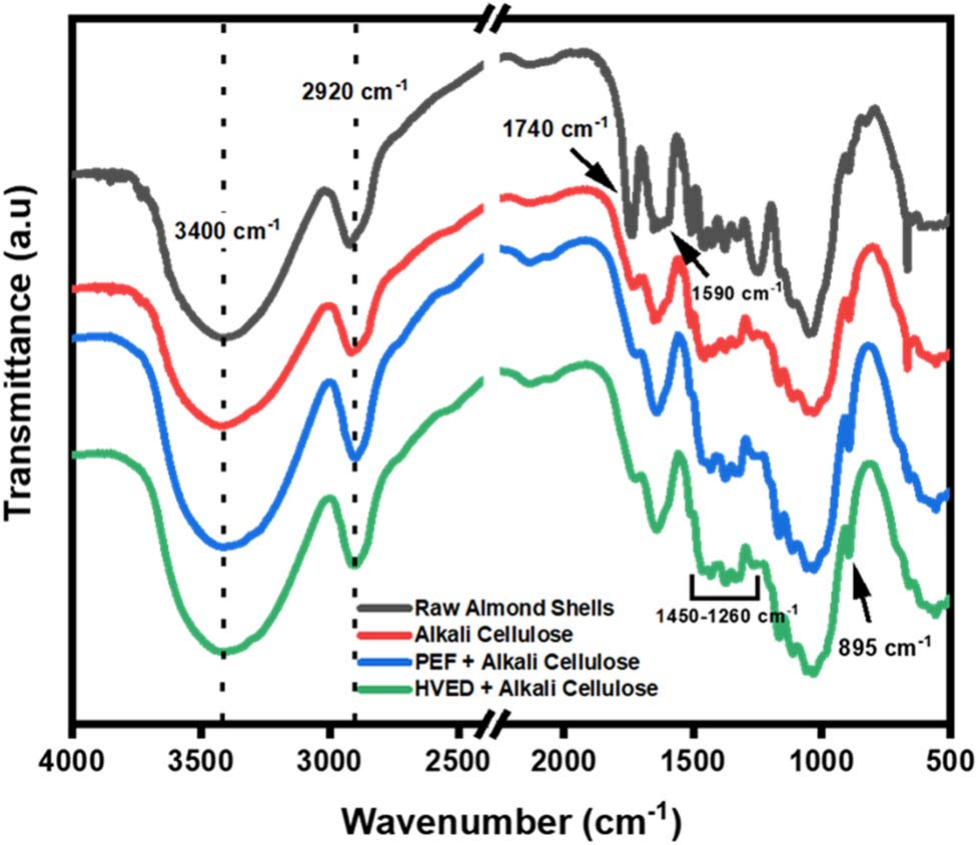
FTIR spectral analysis of raw almond shells and cellulose under alkali treatment, assisted by PEF, and HVED conditions.

Fig. 5 presents the FTIR spectra of raw almond shells (RAS) alongside cellulose extracted using alkali treatment (AC), PEF combined with alkali (PAC), and HVED combined with alkali (HAC). The spectrum of the raw material displays characteristic peaks of lignocellulosic biomass, including a broad band around 3400 cm^−1^, corresponding to O–H stretching vibrations from hydroxyl-rich biopolymers such as cellulose, hemicellulose, and lignin.^37,38^ Peaks in the 2920 cm^−1^ range are attributed to C–H stretching vibrations in methyl, methylene, and methoxyl groups.^37,38^ Notable spectral differences emerge between the raw material and extracted cellulose samples. In all treated celluloses, the absence of the bands around 1740 cm^−1^, associated with the C=O stretching of hemicellulose,^38,39^ and the emergence of a distinct peak at 895 cm^−1^, characteristic of β-glycosidic linkages in cellulose, confirms the effective removal of non-cellulosic components.^37,39^ Alkali treatment alone clearly shows these transitions, while the PAC (PEF) and HAC (HVED) spectra exhibit comparable trends, suggesting that both electrical-assisted methods also facilitate efficient delignification and hemicellulose removal. These structural modifications are critical for adhesive performance, as the enhanced purity and preserved cellulose backbone may contribute to improved interfacial bonding and stability within bio-based adhesive formulations.

**Fig. 6:**
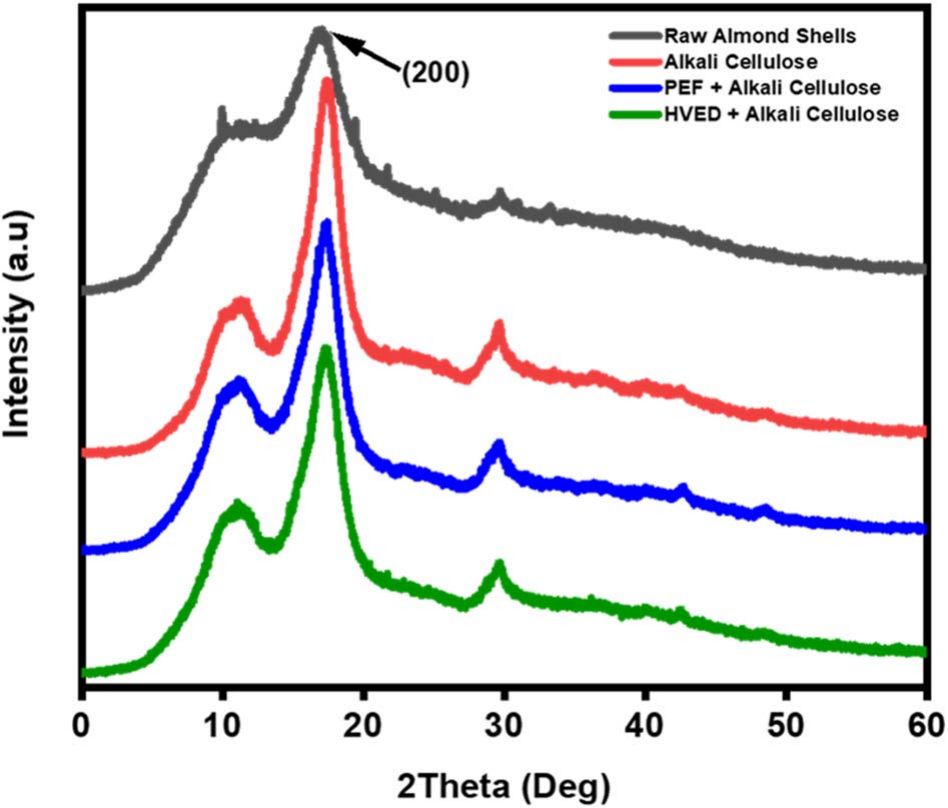
XRD diffraction patterns of raw almond shells and cellulose extracted under alkali treatment, assisted by PEF, and HVED conditions.

The crystalline structure and crystallinity degree of the extracted cellulose samples were assessed using X-ray diffraction (XRD), with the diffraction patterns presented in Fig. 6 and the corresponding crystallinity index (CrI) values summarized in Table 2. All samples exhibit three characteristic diffraction peaks at approximately 2*θ* = 16.4°, 22.3°, and 34.6°, corresponding to the (110), (200), and (004) crystallographic planes of cellulose I, respectively. These peak positions and intensities are consistent with the native cellulose I polymorph, as reported in the literature,^40,41^ indicating that the crystal structure remains largely unaffected by the different extraction methods. Importantly, the preservation of the cellulose I structure across all treatments reinforces the classification of almond shells as a lignocellulosic biomass and demonstrates that the applied pretreatments, whether alkali alone or combined with electrical techniques such as PEF or HVED, do not induce polymorphic transformation.^28,39^ Raw almond shells exhibit a broad and less intense diffraction peak, corresponding to a lower crystallinity index of 37.9%. This lower value reflects the heterogeneous nature of the raw biomass, which contains amorphous components such as lignin and hemicellulose.^37,38^ In contrast, all treated cellulose samples show sharper and more intense peaks, indicating an increase in crystalline order. The CrI values of AC (58.9%), PAC (58.9%), and HAC (58.8%) are nearly identical, demonstrating that while chemical delignification effectively enhances cellulose crystallinity, the additional application of electrical pretreatments (PEF or HVED) does not significantly alter the crystalline structure. These results suggest that the main driver of crystallinity enhancement is the alkali treatment, which efficiently removes amorphous regions. From an application perspective, higher cellulose crystallinity is beneficial for bio-based wood adhesives, as it contributes to improved mechanical strength, dimensional stability, and water resistance, key parameters for enhancing adhesive performance in wood composites.

**Table 2:** Crystallinity index and thermal characteristics of raw almond shells and cellulose samples obtained through alkali extraction, assisted by PEF and HVED processes

Samples	XRD	TGA/DTG	
	CrI	*T*_onset_	*T*_max_
	%	°C	°C
RAS	37.9	258	258
AC	58.9	293	293
PAC	58.9	301	335
HAC	58.8	303	333

##### 3.2.2.3 Thermal stability analysis

**Fig. 7:**
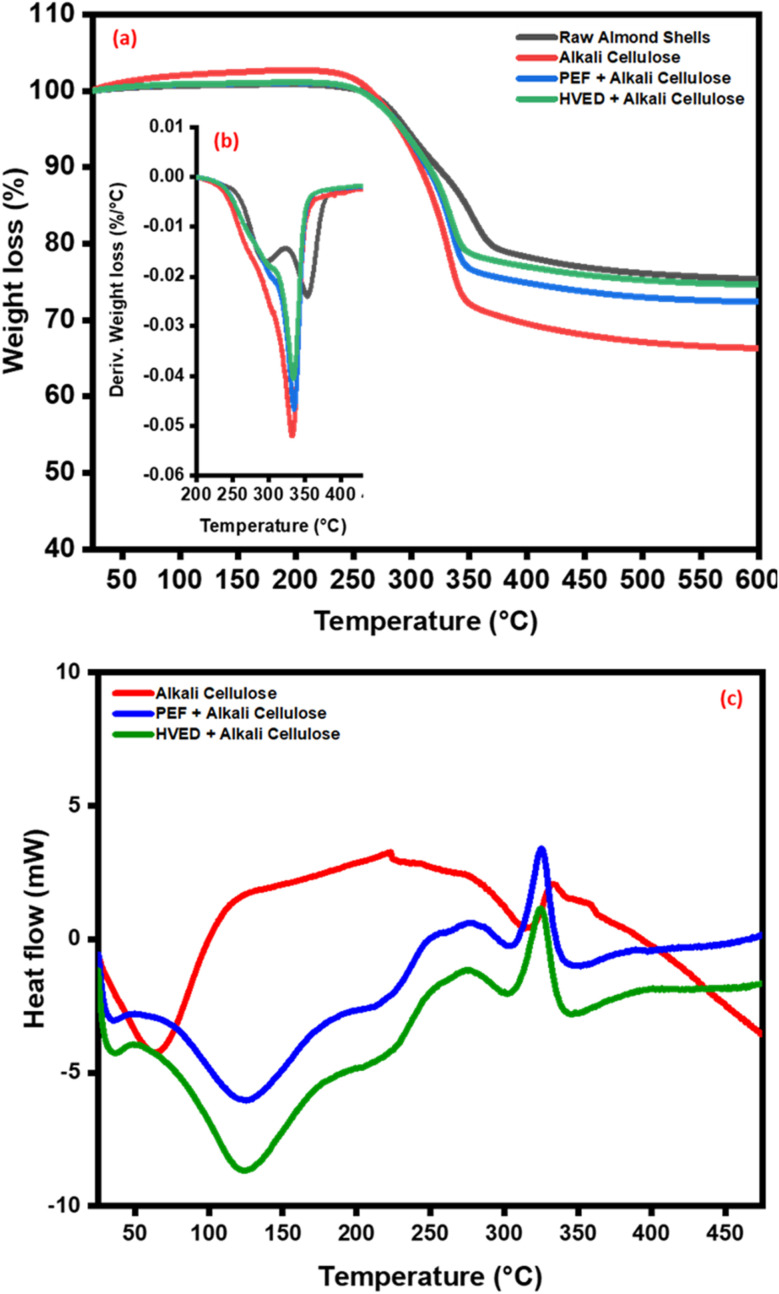
TGA (a), DTG (b), and DSC (c) thermograms of raw almond shells and cellulose extracted under alkali treatment, assisted by PEF, and HVED conditions.

The TGA/DTG results (Fig. 7a, b and Table 2) show that the extracted cellulose exhibits higher onset (*T*_onset_) and maximum (*T*_max_) decomposition temperatures, indicating improved thermal stability. Among the methods, cellulose samples exhibited significantly higher *T*_onset_ values (288–303 °C) compared to raw almond shells (258 °C), indicating enhanced thermal stability after pretreatment. Among these, cellulose obtained with PEF and HVED showed the highest *T*_onset_ values (301 °C and 303 °C, respectively), reflecting improved structural integrity, likely due to higher crystallinity. These results confirm that the pretreatment not only influences yield and structure but also governs thermal performance, with cellulose being more thermally stable than lignin.^42,43^ HVED and PEF emerge as particularly effective techniques for enhancing the thermal stability of cellulose^23,44^

DSC analysis (Fig. 7c) further elucidates the thermal behavior of the extracted materials by measuring heat flow during phase transitions and decomposition reactions. The raw powder sample shows a gradual increase in heat flow with broad transitions, whereas pretreated cellulose samples exhibit characteristic endothermic and exothermic peaks around 300–350 °C, suggesting enhanced crystallinity and improved thermal stability compared to the raw material. Among them, PAC and HAC show sharper peaks, implying more ordered structures, while AC displays broader transitions, indicating partial disruption of crystallinity. This contrast in the thermal behavior of the cellulose emphasizes their distinct chemical and structural properties.^45,46^

The thermal stability trends observed in TGA/DTG and DSC analyses align with structural changes identified through FTIR and XRD. The FTIR spectra revealed the removal of non-cellulosic components and the exposure of cellulose functional groups, as evidenced by the disappearance or shifting of characteristic peaks, particularly in the HAC and AC samples. This structural purification correlates with the increased *T*_onset_ and *T*_max_ values, indicating a more thermally stable cellulose product. Similarly, XRD analysis demonstrated increased crystallinity for the extracted cellulose, with HAC achieving the highest crystallinity index. The elevated crystallinity, indicative of the crystalline cellulose I structure, explains the enhanced thermal resistance observed in TGA/DTG results, as highly crystalline materials typically exhibit superior thermal stability. The improved thermal stability of cellulose is particularly advantageous for adhesive applications, where resilience to high temperatures during processing and usage is essential.^47^

The thermal stability trends observed in TGA/DTG and DSC analyses align closely with the structural changes identified through FTIR and XRD, where the removal of non-cellulosic components and the exposure of cellulose functional groups correlate with increased *T*_onset_ and *T*_max_ values. While XRD analysis confirmed a higher crystallinity index for HAC and PAC samples, indicative of a stable cellulose I structure, this improved thermal resistance should be interpreted as a synergistic result of several variables rather than crystallinity alone. Beyond crystalline integrity, the superior thermal performance of pretreated samples reflects the preservation of high-molecular-weight cellulose chains and the efficient removal of thermally labile hemicelluloses, alongside the potential presence of residual lignin whose complex aromatic structure provides a robust thermal barrier. Consequently, the thermal robustness of these fibers results from an optimized lignocellulosic composition that ensures the necessary resilience for bio-based adhesive applications during high-temperature hot-pressing and industrial usage.

##### 3.2.2.4 Morphology analysis

**Fig. 8:**
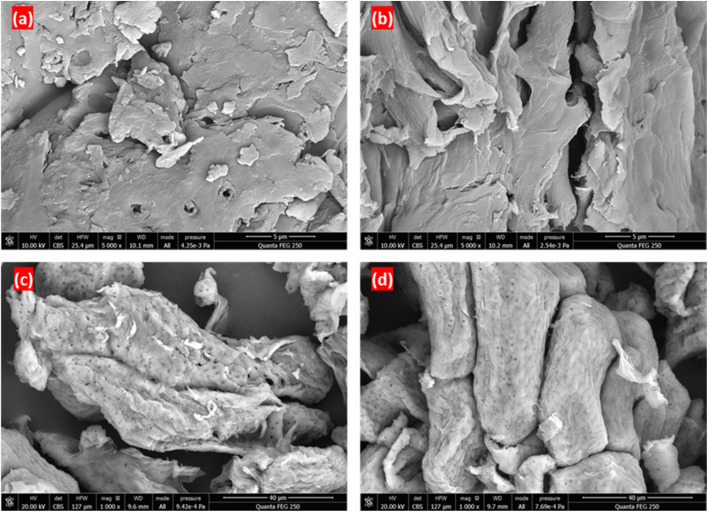
SEM micrographs of raw almond shells (a), cellulose extracted using alkali treatment (b), PEF combined with alkali (c), and HVED combined with alkali (d) from almond shells.

The SEM micrographs (Fig. 8) of raw almond shells (RAS) and cellulose extracted using different treatments, alkali (AC), pulsed electric fields plus alkali (PAC), and high-voltage electrical discharges plus alkali (HAC), reveal significant morphological differences resulting from the applied pretreatment methods. Image (a), corresponding to RAS, exhibits a dense and compact surface structure with minimal porosity, indicating the presence of a rigid lignocellulosic matrix rich in lignin and hemicellulose.^37,38^ In contrast, the surface of the alkali-treated cellulose (b) shows a more fibrillated and disrupted morphology, reflecting the partial removal of non-cellulosic components and the exposure of cellulose fibrils.^38,41^ Image (c), representing PAC, displays a more loosened and porous structure, with disaggregated fiber bundles and increased surface roughness. This suggests enhanced delignification and hemicellulose removal due to electroporation effects induced by PEF, facilitating alkali penetration. Similarly, the HAC (d) presents a highly disrupted and fibrillated surface, indicating even more intense structural damage and efficient matrix disintegration caused by shockwaves, plasma channels, and localized heating associated with HVED. Overall, SEM analysis confirms that electrical-assisted pretreatments (PEF and HVED) promote greater disintegration of the almond shell structure compared to alkali treatment alone. This structural modification is favorable for improving cellulose dispersion and interfacial bonding in adhesive formulations, potentially enhancing the performance of cellulose–cornstarch–mimosa tannin adhesives in wood-based applications.

### 3.3 Particleboard characterization

The bonding mechanism between the starch–tannin–cellulose adhesive and wood particles is mainly governed by interfacial hydrogen bonding and physical entanglement. The abundant hydroxyl groups in starch and cellulose interact with hydroxyl and phenolic groups of wood constituents, while tannin molecules further enhance interfacial adhesion through multiple hydrogen-bonding sites. Upon hot pressing, these interactions promote the formation of a dense, interconnected adhesive network, resulting in improved interfacial contact and mechanical integrity of the particleboard.

#### 3.3.1 Mechanical characteristics of particleboards

**Fig. 9:**
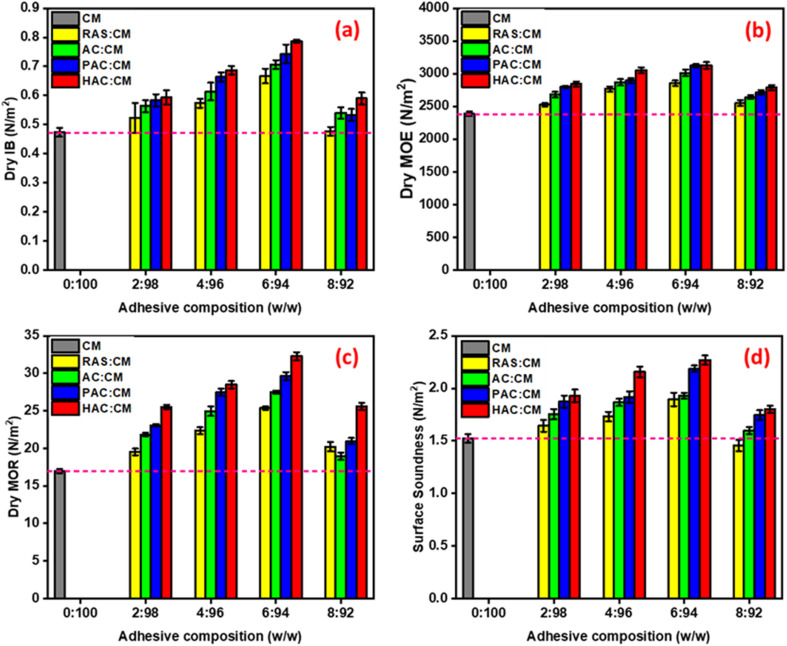
Variation in (a) dry internal bond, (b) dry modulus of elasticity, (c) dry modulus of rupture, and (d) surface soundness of cornstarch–mimosa tannin adhesives formulated with cellulose obtained under alkali treatment, assisted by PEF, and HVED conditions.

The results presented in Fig. 9 clearly demonstrate that incorporating almond shell-derived cellulose into the cornstarch–mimosa tannin adhesive formulation significantly improves the mechanical performance of the resulting particleboards. Across all treatments, (a) dry internal bond, (b) dry modulus of elasticity, (c) dry modulus of rupture, and (d) surface soundness progressively increased with cellulose loading up to 6 wt%, followed by a slight decline at 8 wt%. This trend highlights the reinforcing capability of cellulose, which enhances the cohesive strength of the adhesive matrix through extensive hydrogen bonding and increased polymer–filler interactions. Among the treatments, HAC consistently produced the highest mechanical values, indicating that HVED-assisted extraction yields a more fibrillated and reactive cellulose structure capable of forming strong interfacial networks with starch and tannin.^48^ Notably, particleboards prepared with 6 wt% HAC–CM exceeded the EN 312 (P4) requirements in all mechanical categories, confirming the high reinforcing effect of HVED-extracted cellulose.

However, a consistent decline in mechanical values was observed at a loading of 8 wt%. This performance drop is likely governed by two synergistic mechanisms: fiber agglomeration and impaired adhesive fluidity. At higher concentrations, the high surface energy and hydroxyl density of the cellulose fibers promote self-association and entanglement, leading to the formation of clusters that act as structural defects rather than reinforcing bridges.^49,50^ Furthermore, the excessive fiber loading significantly increases the viscosity of the adhesive, which reduces its wetting efficiency on the wood particles. This leads to a discontinuous adhesive film and poor interfacial contact during the hot-pressing process, ultimately weakening the overall cohesive strength of the particleboard.^51,52^

Furthermore, the substantial improvements observed in modulus of elasticity and modulus of rupture for PAC-CM and HAC-CM adhesives emphasize the role of electrical-assisted extraction in enhancing cellulose's structural integrity and surface reactivity. The superior performance of HAC-CM reflects the combination of high crystallinity, increased fibrillation, and enhanced thermal stability previously identified in Section 3.2, which collectively promote efficient stress transfer within the composite. Surface soundness also followed the same enhancement pattern, demonstrating that cellulose reinforcement improves the interfacial adhesion between surface layers and the core of the particleboard. Overall, the mechanical behavior shown in Fig. 9 confirms that cellulose obtained *via* HVED provides the most effective strengthening effect, enabling the development of high-performance, formaldehyde-free particleboards that meet and surpass industrial standards.

The mechanical performance data presented in Table 3 confirm the substantial reinforcing effect of almond shell-derived cellulose on CM-based particleboards. Across all extraction methods, the addition of 2–6 wt% cellulose or raw almond shell powder resulted in systematic increases in internal bond, modulus of elasticity, modulus of rupture, and surface soundness, compared to the unmodified CM adhesive. This improvement is attributed to the ability of cellulose microfibrils to form strong intermolecular hydrogen bonds with starch and tannin, thereby enhancing the cohesive strength of the adhesive network and improving load transfer within the composite structure.^53,54^ The mechanical properties peaked consistently at a cellulose loading of 6 wt%, beyond which all formulations experienced a decrease, likely due to filler aggregation and reduced polymer mobility at higher solid contents. Notably, even the lowest cellulose loadings (2 wt%) provided values well above the EN 312 requirements for P2 and P4 boards, underscoring the effectiveness of cellulose integration.

**Table 3:** Mechanical properties of particleboards compared to European standard requirements (EN) and control adhesive

Adhesives	Content (w/w)	Dry IB (MPa)	Dry MOE (MPa)	Dry MOR (MPa)	SS (MPa)
CM	0 : 100	0.47 ± 0.01	2386 ± 34	16.95 ± 0.29	1.52 ± 0.04
RAS : CM	2 : 98	0.52 ± 0.05	2524 ± 24	19.51 ± 0.43	1.64 ± 0.06
	4 : 96	0.57 ± 0.01	2765 ± 40	22.35 ± 0.44	1.73 ± 0.05
	6 : 94	0.67 ± 0.02	2853 ± 41	25.35 ± 0.23	1.89 ± 0.06
	8 : 92	0.48 ± 0.01	2550 ± 42	20.22 ± 0.64	1.45 ± 0.05
AC : CM	2 : 98	0.56 ± 0.02	2679 ± 41	21.82 ± 0.23	1.75 ± 0.05
	4 : 96	0.61 ± 0.03	2869 ± 48	24.94 ± 0.64	1.86 ± 0.03
	6 : 94	0.71 ± 0.01	3012 ± 49	27.48 ± 0.23	1.93 ± 0.03
	8 : 92	0.54 ± 0.02	2642 ± 29	18.95 ± 0.48	1.59 ± 0.03
PAC : CM	2 : 98	0.58 ± 0.02	2797 ± 15	23.06 ± 0.17	1.87 ± 0.06
	4 : 96	0.66 ± 0.01	2894 ± 40	27.46 ± 0.48	1.91 ± 0.05
	6 : 94	0.74 ± 0.03	3123 ± 24	29.64 ± 0.54	2.18 ± 0.03
	8 : 92	0.53 ± 0.02	2713 ± 34	20.95 ± 0.41	1.74 ± 0.04
HAC : CM	2 : 98	0.59 ± 0.02	2840 ± 40	25.53 ± 0.28	1.92 ± 0.06
	4 : 96	0.69 ± 0.01	3052 ± 46	28.47 ± 0.48	2.15 ± 0.05
	6 : 94	0.79 ± 0.01	3125 ± 50	32.27 ± 0.55	2.27 ± 0.04
	8 : 92	0.59 ± 0.02	2786 ± 40	25.59 ± 0.46	1.80 ± 0.04
Requirements EN 312 (P2 boards)		>0.35	>1600	>13	>1
Requirements EN 312 (P4 boards)		>0.35	>2300	>15	>1

Among the cellulose sources, HAC produced the highest mechanical performance at every loading level, culminating in an internal bond of 0.79 MPa, modulus of elasticity of 3125 MPa, modulus of rupture of 32.27 MPa, and surface soundness of 2.27 MPa at 6 wt%, the highest values observed in this study. These results align with the superior crystallinity, thermal stability, and fibrillation of HVED-extracted cellulose discussed previously, demonstrating its exceptional reinforcing capability. PAC and AC also significantly outperformed RAS powders and the control adhesive, with PAC-CM approaching HAC-CM values due to the enhanced surface accessibility and microstructural damage imparted by PEF treatment. Overall, all optimized cellulose-containing adhesives surpassed the EN 312 (P4) thresholds, confirming that electrical-assisted cellulose extraction enables the development of high-strength, formaldehyde-free particleboards suitable for demanding structural applications.

#### 3.3.2 Physical properties of particleboards

**Fig. 10:**
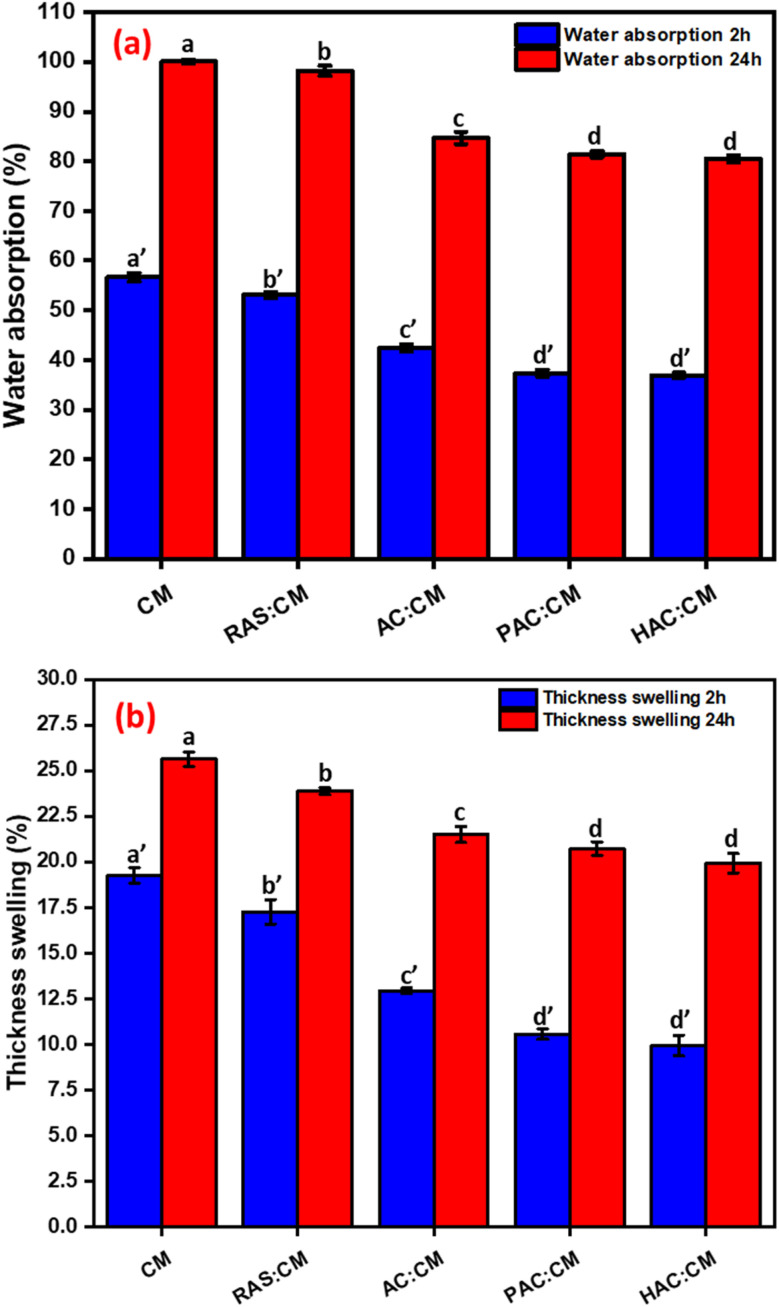
Water absorption (a) and thickness swelling (b) measured after 2 h and 24 h of immersion for cornstarch–mimosa tannin adhesive composites reinforced with cellulose obtained under alkali treatment, assisted by PEF, and HVED conditions.

Fig. 10 illustrates the water absorption (a) and thickness swelling (b) of cornstarch–mimosa tannin (CM) adhesive composites reinforced with cellulose obtained *via* different electrical-assisted extraction methods after 2 h and 24 h of water immersion. A clear improvement in water resistance was observed with the incorporation of cellulose, particularly with cellulose extracted using PEF and HVED methods. The control CM composite exhibited the highest water absorption after 24 h immersion, indicating its inherent hydrophilic nature and susceptibility to moisture uptake due to the abundant hydroxyl groups in starch and tannin. In contrast, cellulose-modified adhesives showed a noticeable reduction in water absorption, with HAC-CM showing the lowest value. This reduction can be attributed to the densification of the composite matrix and the formation of stronger hydrogen-bonded and crosslinked networks between cellulose microfibrils and the adhesive polymers, which hinder water diffusion.^55^ Moreover, the presence of well-dispersed cellulose increased the tortuosity of water penetration pathways, contributing to the lower equilibrium moisture content. The smaller difference between 2 h and 24 h absorption in HAC-CM also suggests that equilibrium was achieved faster, reflecting a more stable and less hydrophilic matrix.

A similar trend was observed for thickness swelling (Fig. 10b), where cellulose reinforcement markedly reduced dimensional instability after water immersion. The unmodified CM composite exhibited the highest swelling values, confirming that excessive water uptake led to significant internal stress and expansion. However, as cellulose was introduced, especially through PAC and HAC, the thickness swelling decreased substantially for both 2 h and 24 h immersion periods. This enhancement results from the strong interfacial adhesion between cellulose and the polymeric network, which restricts the relaxation and re-expansion of the adhesive matrix upon water absorption. The high aspect ratio and surface functionality of cellulose promote compact packing and efficient stress distribution, thereby minimizing structural deformation. Among all formulations, HAC-CM showed the best dimensional stability, aligning with its superior mechanical performance (as observed in Fig. 9 and Table 3). These results collectively confirm that cellulose extracted *via* HVED techniques imparts hydrophobic character, enhances internal bonding, and improves moisture durability of the cornstarch–mimosa tannin adhesives, making them more suitable for humid or load-bearing applications in particleboard production.

#### 3.3.3 Comparative performance analysis

Table 4 highlights clear performance and sustainability trade-offs between the bio-based starch–tannin–cellulose adhesive and conventional UF and PF systems. Unlike UF and PF resins, which release formaldehyde at low to high levels and are associated with carcinogenic or corrosive health risks, the bio-based adhesive exhibits zero formaldehyde emissions (E0) and non-toxic handling due to its fully plant-derived composition.^56,57^ While UF remains economically attractive as an industrial standard, its poor moisture resistance limits durability, whereas PF offers excellent water resistance at higher material cost and toxicity.^58,59^ The bio-based adhesive demonstrates good moisture resistance attributed to tannins and cellulose, combined with a competitive hot-pressing temperature range (180–200 °C) comparable to PF systems, indicating strong potential as a safer and more sustainable alternative for wood-based panel applications.^58,60^

**Table 4:** Bio-based adhesive (this study) *vs.* Industrial adhesives (UF/PF)

Criterion	Starch–tannin–cellulose adhesive	Urea–formaldehyde (UF) adhesive	Phenol–formaldehyde (PF) adhesive	References
Formaldehyde emissions	None (E0)	High (class E1 or E2)	Low but present	56 and 57
Origin of components	100% natural (plant-based)	100% petrochemical	100% petrochemical	58 and 61
Toxicity/health	Non-toxic, safe handling	Carcinogenic	Toxic and corrosive	62 and 63
Cost of materials	Low (by-products recovered)	Very low (industry standard)	Higher than UF	62 and 63
Moisture resistance	Good (thanks to tannins/cellulose)	Low (easy hydrolysis)	Excellent (outdoor use)	58 and 59
Forecast temperature	180–200 °C	160–180 °C	180–220 °C	58 and 60

The performance of the developed cellulose-reinforced cornstarch–tannin adhesive was benchmarked against prominent commercial formaldehyde-free systems, such as soy protein and lignin-based adhesives. Particleboards bonded with soy protein have historically achieved dry shear strengths comparable to M-2 grade standards, while lignin-based systems often meet international norms for exterior applications. In comparison, our formulation exhibits high mechanical competitiveness, satisfying the EN 312 (P2) European norms for interior fittings, similar to the results reported for standard cornstarch-tannin adhesives.^64^ Critically, the integration of Electrical-Assisted extracted cellulose addresses the typical moisture-sensitivity issues seen in many bio-based resins, yielding thickness swelling and water absorption values that rival lignin-based benchmarks.^65^ While traditional adhesives remain prevalent due to cost-effectiveness, the superior mechanical-hydrophobic balance achieved in this study highlights the potential of the cornstarch–tannin–cellulose system as a high-performance, sustainable alternative in the wood composite market.

## 4 Conclusions

This study demonstrated the effectiveness of electrical-assisted extraction methods, specifically PEF and HVED, in improving the structural and functional characteristics of cellulose extracted from almond shells, a lignocellulosic agricultural residue. Compared to conventional alkali extraction, electrical pretreatments significantly enhanced cell wall disruption, delignification, and fibrillation, leading in improved cellulose purity and surface reactivity while maintaining the native cellulose I crystalline structure. Characterization through FTIR, XRD, TGA/DTG, DSC, and SEM confirmed that PEF- and HVED-assisted extractions increased crystallinity (≈59%), thermal stability (*T*_onset_ ≈ 303 °C), and morphological homogeneity. These results highlight that combining moderate alkaline treatment with electrical-assisted technologies offers an efficient and eco-friendly route for cellulose recovery, minimizing chemical usage and processing time.

Overall, the mechanical evaluation confirms that incorporating cellulose, especially that extracted through HVED, substantially strengthens CM-based adhesives and the resulting particleboards. The optimized formulations at 6 wt% cellulose consistently surpassed EN 312 (P4) requirements, demonstrating enhanced bonding, stiffness, and structural reliability. At an optimal loading of 6 wt%, cellulose obtained *via* HVED treatment (HAC) delivered the highest performance, with internal bond (0.79 MPa), modulus of rupture (32.27 MPa), and modulus of elasticity (3125 MPa) surpassing both unmodified adhesives and EN 312 (P4) standards. These improvements were attributed to the microfibrillated structure and abundant hydroxyl groups of the HVED-derived cellulose, which promoted extensive hydrogen bonding and network crosslinking. Additionally, particleboards reinforced with HAC-CM adhesives exhibited superior water resistance and dimensional stability, confirming that electrical-assisted cellulose enhances both dry and wet performance.

Overall, this research provides a sustainable and high-performance alternative to formaldehyde-based adhesives. The synergy between green extraction techniques and bio-based adhesive formulations demonstrates a circular valorization approach for agricultural by-products. HVED-assisted cellulose extraction not only improves adhesive strength and water resistance but also aligns with the principles of energy efficiency, reduced environmental impact, and resource recovery.

Despite the high mechanical performance achieved in this study, certain limitations remain to be addressed for industrial scaling. The current evaluation focused on immediate properties, whereas the long-term durability and aging resistance of these bio-based particleboards under fluctuating environmental conditions require further investigation. Additionally, while electrical-assisted extraction reduces chemical consumption, a comprehensive life cycle assessment and a cost-benefit analysis regarding the initial capital investment for high-voltage equipment are necessary to fully establish its economic feasibility. Future work will focus on pilot-scale production trials and the incorporation of natural anti-fungal agents to enhance the biological resistance of the panels, ensuring their long-term stability and competitiveness in the global furniture and construction markets.

## Author contributions

Yassine El Khayat Driaa: conceptualization, formal analysis, data curation, investigation, writing – original draft. Hafida MAARIR: formal analysis, writing – original draft. Nabil GRIMI: supervision, visualization, validation. Nadia BOUSSETTA: supervision, visualization, validation. Amine MOUBARIK: supervision, visualization, validation.

## Conflicts of interest

The authors declare no competing interests.

## Abbreviations

AC: Alkali cellulose
AS: Almond shells
AT: Alkali treatment
CM: Cornstarch–tannin mimosa
CrI: Crystallinity index
DSC: Differential scanning calorimetry
DTG: Derivative thermogravimetry
FTIR: Fourier transform infrared spectroscopy
HAC: HVED + alkali cellulose
HVED: High-voltage electrical discharges
IB: Internal bond strength
MOE: Modulus of elasticity
MOR: Modulus of rupture
NaClO_2_: Sodium chlorite
NaOH: Sodium hydroxide
PAC: PEF + alkali cellulose
PEF: Pulsed electric fields
RAS: Raw almond shells
SEM: Scanning electron microscopy
SS: Surface soundness
TGA: Thermogravimetric analysis
*T*_max_
: Maximum degradation temperature
*T*_onset_
: Onset degradation temperature
XRD: X-ray diffraction

## Data Availability

The data that support the findings of this study are available from the corresponding author upon reasonable request.
